# A treatment planning study comparing IMRT techniques and cyber knife for stereotactic body radiotherapy of low-risk prostate carcinoma

**DOI:** 10.1186/s13014-019-1353-6

**Published:** 2019-08-09

**Authors:** Sergiu Scobioala, Christopher Kittel, Khaled Elsayad, Kai Kroeger, Michael Oertel, Laith Samhouri, Uwe Haverkamp, Hans Theodor Eich

**Affiliations:** Department of Radiation Oncology of the University Hospital Muenster, Albert-Schweitzer-Campus 1, Gebäude 1, 48149 Muenster, Germany

**Keywords:** Prostate carcinoma, Stereotactic radiotherapy, Intensity-modulated radiotherapy, Cyber knife, Dosimetric indices, Normal tissue complication probability

## Abstract

**Purpose:**

Comparing radiation treatment plans by using the same safety margins and dose objectives for all techniques, to ascertain the optimal radiation technique for the stereotactic body radiotherapy (SBRT) of low-risk prostate cancer.

**Material and methods:**

Treatment plans for 27 randomly selected patients were compared using intensity-modulated (IMRT) techniques as Sliding Window (SW), volumetric modulated arc therapy (VMAT), and helical tomotherapy (HT), as well as Cyber Knife (CK) system. The target dose was calculated to 36.25 Gy delivered in five fractions over 1 week. Dosimetric indices for target volume and organs at risk (OAR) as well as normal tissue complication probability (NTCP) of late rectal and urinary bladder toxicities were analyzed.

**Results:**

The CK provided lower homogeneity in the target volume, but higher values for most of the conformity indices compared to the IMRT approaches. The SW demonstrated superior rectum sparing at medium-to-high dose range (V18 Gy - V32.6 Gy) compared to other techniques (*p* < 0.05). The whole urinary bladder experienced the best shielding by SW and VMAT at the medium dose (V18 Gy, *p* < 0.05 versus CK), however we obtained no relevant differences between techniques at the high dose. Generally, the CK demonstrated significantly superior rectum and bladder exposure at V18 Gy as compared to HT, SW, and VMAT. For the rectum, mean NTCP values were significantly superior for HT (NTCP = 2.3%, *p* < 0.05), and for urinary bladder, the NTCP showed no significant advantages for any technique.

**Conclusion:**

No absolute dosimetric advantage was revealed to choose between CK or IMRT techniques for the SBRT of low-grade prostate cancer. Using the same safety margins and dose objectives, IMRT techniques demonstrated superior sparing of the rectum and bladder at a medium dose compared to CK. Comparing different IMRT approaches SW displayed superior rectum sparing at a medium-to-high dose range, whereas both SW and RA revealed superior bladder sparing compared to HT. HT demonstrated a significantly lower NTCP outcome compared to CK or IMRT techniques regarding the rectum. Radiation plans can be optimized further by an individual modification of dose objectives independent of the treatment plan strategy.

**Electronic supplementary material:**

The online version of this article (10.1186/s13014-019-1353-6) contains supplementary material, which is available to authorized users.

## Introduction

Different radiotherapy techniques, as well as fractionation regimens, are currently used for localized prostate cancer. A conventionally-fractionated intensity modulated radiotherapy (IMRT) is the most frequently applied treatment modality in the case of prostate cancer. One retrospective analysis evaluated the standard fractionation versus hypofractionation regimens. This analysis suggests that the α/β value for prostate cancer is 1.4 Gy (0.9–2.2) regardless of risk status [1]. Furthermore, hypofractionated radiotherapy (RT) may be radiobiologically favorable in the treatment of prostate cancer due to potentially greater sensitivity of high fraction dose [2–4]. Previous studies of moderate hypofractionated RT (fraction dose between 2.5 Gy and 3.5 Gy) showed better disease local control, as well as similar toxicity rate compared to conventionally-fractionated RT [5–7]. Different radiation techniques, additionally, may be applied for stereotactic body RT (SBRT) in order to deliver a large fraction dose to the prostate. RT modality as provided by the robot-assisted technique Cyber Knife® (CK), which can deliver such radiation within a high fraction dose, is currently witnessing increased usage in the treatment of prostate cancer with low-to-intermediate risk [2, 8–12]. All IMRT techniques, especially rotational approaches as helical tomotherapy (HT) and volumetric modulated arc therapy (VMAT), can potentially deliver a high daily fraction, thus, achieving the treatment plans with high conformity and reducing the dose delivered to the surrounding healthy tissue. In the series of studies a good dosimetric quality for several SBRT techniques for the treatment of localized prostate cancer was demonstrated [13–17]. In this study, we performed a comparative statistical analysis of dosimetric parameters between Sliding Window (SW), HT, VMAT, and CK for the SBRT in patients with low-risk prostate cancer. It is the first planning study that, specifically, uses the Lyman-Kutcher-Burman (LKB) model in the analysis of late rectal and urinary toxicities following prostate SBRT based on estimation of NTCP parameters.

## Material and methods

### Patients

The treatment plans were generated for 27 randomly selected patients with low-risk prostate cancer who underwent definitive HT. The patient and tumor characteristics are presented in Additional file 1: Table S1 The mean age of study patients was 68 years (range 58 to 77 years). Patient selection criteria was based on the pathology of proven low-risk prostate cancer; PSA less than 10 ng/mL, Gleason Score 6 - 7a, and T1c - T2a per national comprehensive cancer network (NCCN) criteria [18].

### Plan generation and analysis of dosimetric parameters

The databases of a thin-cut 3 mm CT scans were fused with 1.5 T MRI. The prescription dose was 5 fractions of 7.25 Gy to the 80% isodose. The corresponding dose was 36.25 Gy, with at least 98% coverage of the PTV. The potential maximum coverage of 100% of the prescribed dose covered 2% of PTV. This corresponds to a biological equivalent dose (BED) of approximately 90 Gy by the α/β value of 1.5 for prostate, 3 for rectum and 6 for urinary bladder [3, 4, 19–21]. Similar dose objectives adapted to the criteria of ICRU 83 were used by the planning for VMAT, SW, HT, as well as for CK.

The clinical target volume (CTV) and planning target volume (PTV) were defined according to Boehmer et al. [22]. The CTV included the prostate directly without seminal vesicles. A 3 mm margin in the dorsal direction and 5 mm margin in ventral and lateral directions was further added for PTV delineation*.* The prescription dose was limited to ≥80% in order to restrict maximum dose to the prostatic urethra with 110% of the prescription dose. To assess the PTV coverage, we have used the following parameters: PTVV80% – percentage volume of PTV receiving 36.25 Gy; PTVD2% (max, Gy) and PTVD98% (min, Gy) - maximum and mean dose to the PTV. The dose-volume objectives for the OAR are presented in Table 1. The dose constraints for OAR, furthermore, had met qualifying criteria per the Quantitative Analyses of Normal Tissue Effects in the Clinic (QUANTEC) reports (Table 1) [8, 9, 13, 14, 19, 20]. The patient-averaged dose-volume histograms (DVH) were statistically analyzed for PTV and OAR.

**Table 1:** Organ at risk dose constraints

Organ		
Rectum	V18 Gy	< 50%
	V29 Gy	< 20%
	V32.6 Gy	< 10%
	V36.25 Gy	< 5%
Urinary bladder	V18 Gy	< 40%
	V36.25 Gy	< 10%
Femoral heads	V14.5 Gy	< 5%

*Abbreviations*: *Vx* Percent of OAR volume exposed to certain radiation dose

Based on the recommendations of the ICRU 83 reports and several clinical studies, specific conformal indices were used to describe the dose distribution. These indices refer to the PTV volume, partial PTV volume covered by prescribed isodose (PTVpi), and volume treated by prescribed isodose (TVpi) [23]. The homogeneity index characterizes dose homogeneity inside of the PTV and is calculated using the near maximum (D2%), near minimum (D98%), and the mean dose (Dmean): D2% – D98%/Dmean (optimal at 0) [21]. The CN provides complementary information about irradiation of PTV and healthy tissues, and is calculated as PTVpi/PTV x PTVpi/TVpi, demonstrating the optimal results at a value of 1 [24]. The CIICRU was described in the ICRU 62 report and is defined as a quotient of TVpi/PTV (optimal at 1) [25]. The CΔ quantified the radiation exposure of the surrounding healthy tissue and is defined as TVpi - PTVpi/PTV (optimal at 0) [26]. The CΔCOV describes the coverage of the target volume by the prescribed isodose and is calculated as PTVpi/PTV, reflecting ideal PTV coverage for the value of 1 [27].

For this study the plans were generated by more than 3 independent planners. All plans were created for a possible use in the institute. The planning was not affected by previously generated plans because different plans were created by different planners for a patient case.

### Radiation techniques

Seven-field IMRT plans using the SW technique were created on the Eclipse™ 10 treatment planning system [Varian Medical Systems, Palo Alto USA]. All plans were generated for the Varian True Beam linear accelerator (LINAC) using beam energies of 15 MV photons and beam angles of 0°, 51°, 102°, 153°, 204°, 255° and 306°. The VMAT plans were created using the same treatment planning system as for SW. Two incomplete arcs from 200° to 160° for the protection of the posterior rectum wall were used. The plans were created using 15 MV photons with a 0.5 cm leaf width. A maximum dose rate of 600 MU/min as well as MLC motion of 2.5 cm/s was applied. The MLC-leakage was at 1.8%. The HT plans were assessed using Tomo planning system version 5 (Accuray® Inc., Sunnyvale, USA). The plans were created for the High Art HDII HT system that uses a helical slice 6 MV photon beam modulated by 64 binary multileaf collimators. The Multiplan® planning system version 5.2 was used to generate the CK plans (Accuray®, Sunnyvale, USA). The CK robotic arm moves in six different axes with a specific positioning accuracy of 0,2 mm. It delivers a 6 MV photon beam with a dose rate of 850 MU/min. The collimator system consists of 12 fixed cones with a size of 5 mm to 60 mm (at 800 mm SAD).

### NTCP estimation

Lyman’s model of rectal and bladder toxicities was used for estimating NTCPs, which is integrated in the biological evaluation module [28]. Parameters used in the Lyman’s model are presented in Table 2. The dose was recalculated to the conventionally fractionated treatment schedule of 2 Gy per fraction using the α/β value of 3 for rectum, and α/β value of 6 for bladder [29–31]. Lyman’s model is based on a probit function: ${NTCP}_{LKB}=\frac{1}{2\pi }{\int}_{-\infty}^t\mathit{\exp}\left(\frac{-{u}^2}{2}\right) du$, where $t=\frac{D_{eff}-{D}_{50}}{m\bullet {D}_{50}}$ and ${D}_{eff}={\sum}_{i=1}^M\left(\frac{v_i}{V_{ref}}{EQD}_{2,i}^{1/n}\right)2$, where *D*_50_ = dose giving a 50% response probability, m = slope of the response curve, *n* = volume dependence, M = total number of voxels, $\frac{v_i}{V_{ref}}$ = relative volume of voxel compared to reference volume, and *EQD*_2_ = the equivalent dose in voxel when given in 2 Gy fractions.

**Table 2:** Parameters used in the Lyman-Kutcher-Burman model

Parameter	Rectum	Urinary bladder
D50%	80	62
α/β	3	6
n	0.06	0.13
m	0.15	0.11

*Abbreviations*: *D50%* Tolerance dose leading to 50% complications, *n* Volume effect, *m* Steepness of the dose-response curve

### Statistical analysis

The statistical analysis was carried out through SPSS software (SPSS for Windows, Version 24.0). A *p*-value < 0.05 was considered statistically significant. The comparison of dose distribution in the target volume and in the OAR through use of different radiation techniques was examined with the Wilcoxon Test for paired-samples. In a similar way, we tested the homogeneity and conformity of the radiation field in the target volume for different field arrangements. The effect of the variables (techniques) on the NTCP outcome of urinary and rectal toxicities was statistically analyzed by use of Wilcoxon Test.

## Results

The volumes of the prostate and urinary bladder did not exhibit any relevant data concerning dose distribution volumetric differences in analyzed patients. The average of these volumes was 31 cm^3^ (range, 27 cm^3^ to 58 cm^3^) and 300 mL (range, 220 mL to 410 mL), correspondingly. Similarly, the rectal diameter did not significantly vary between patients (median 5 cm, range 3.4 cm to 7.4 cm) (Additional file 1: Table S1.

Conformal indices are summarized in Table 3. HI around 0.2 was received for all techniques. The HT provided a higher homogeneity of dose distribution than in any other technique employing the target volume. This demonstrates a significant advantage compared to CK (*p* = 0.03). Similarly, the coverage index COV appeared inferior for the CK. Other conformity indices (CN, CIICRU_,_ CΔ) revealed superior values for the CK.

**Table 3:** Summary of dosimetric indices averaged from the treatments plans of 27 patients

Index	HT Mean ± SD	SW Mean ± SD	VMAT Mean ± SD	CK Mean ± SD
Homogeneity index, HI	0.18 ± 0.01*	0.21 ± 0.01	0.19 ± 0.01	0.21 ± 0.04*
Conformation number, CN	0.85 ± 0.05	0.81 ± 0.05	0.87 ± 0.03	0.87 ± 0.06
ICRU Conformity index, CIICRU	1.16 ± 0.08	1.22 ± 0.07	1.13 ± 0.04	1.08 ± 0.07
Coverage index, CΔ	0.16 ± 0.08	0.23 ± 0.07	0.14 ± 0.04	0.11 ± 0.06
Coverage index, COV	0.99 ± 0.01	0.99 ± 0.01	0.99 ± 0.01	0.97 ± 0.02

*Abbreviations*: *VMAT* Volumetric Modulated Arc Therapy, *SW* Sliding Window, *HT* Helical tomotherapy, *CK* Cyber Knife, *SD* Standard deviation, *HI* Homogeneity index, *CN* Conformation number, *CIICRU* ICRU conformity index, *C∆* Coverage index, *COV* Coverage index COV; *-values with statistically significant difference (*p* < 0.05)

The time for the planning averaged 2.5 h for CK, 2 h for HT, and 1 h for VMAT and SW. The mean treatment delivery time was significantly higher for CK with 42 min compared to IMRT techniques. Among IMRT approaches, the median treatment time of the HT was significantly higher with 22 min (range: 16–26 min; *p* < 0.05) compared to SW (6 min, range: 4.5–8.2 min) or VMAT (5 min, range: 4–6 min).

**Fig. 1:**
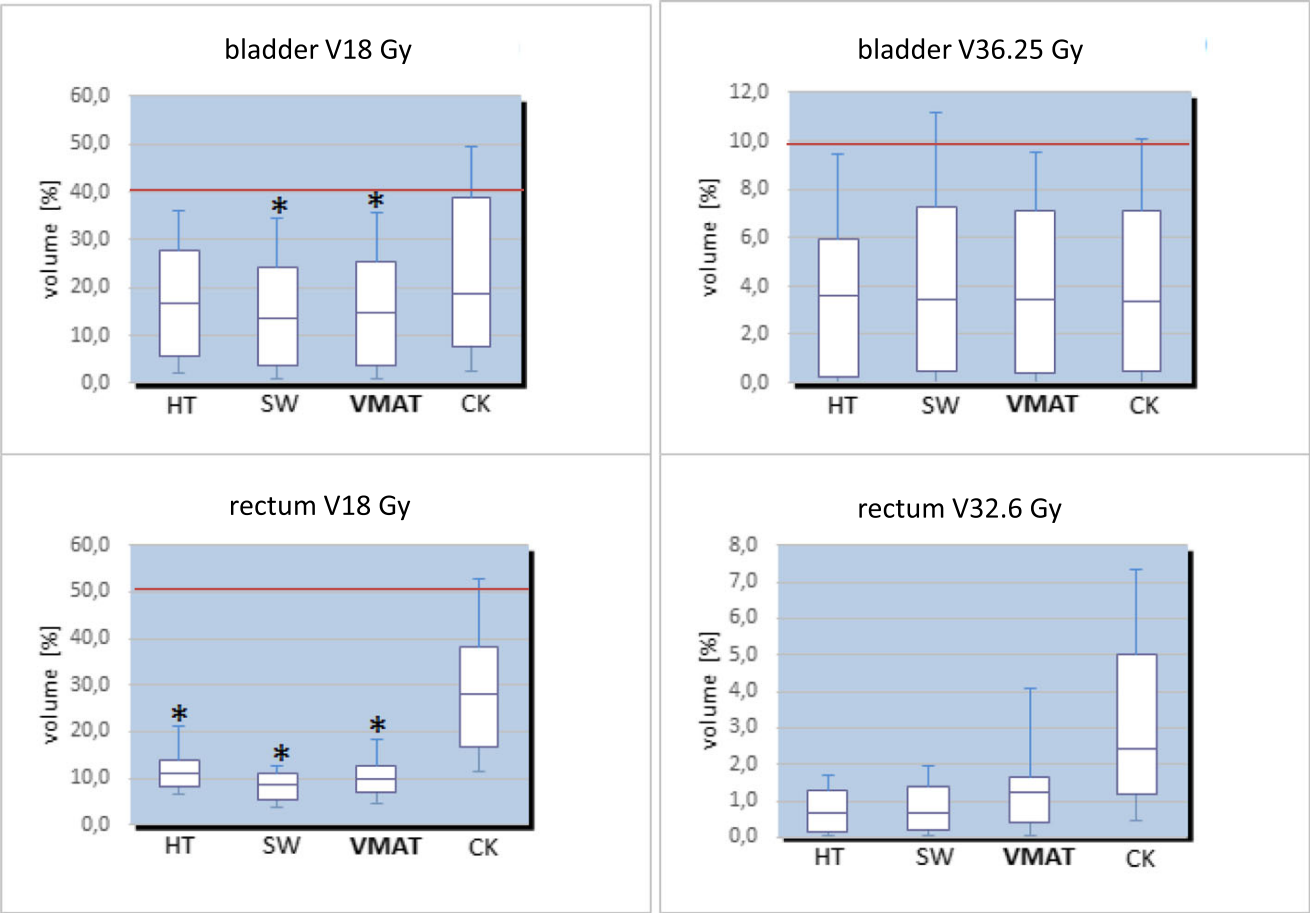
Boxplot demonstrating a difference in the sparing of urinary bladder and rectum by using of different radiation techniques. Red lines represent dose objective levels (level rectum V32.6 Gy <  10% out of scale). * - Significantly (*p* < 0.05) superior sparing for urinary bladder was found for VMAT and SW at V18 Gy vs. CK, and of rectum for Tomo, SW and VMAT at V18 Gy vs. CK. Abbreviations: VMAT – Volumetric Modulated Arc Therapy; SW – Sliding Window; HT– helical tomotherapy; CK – Cyber Knife; Vx – percent of OAR volume exposed to certain radiation dose

**Fig. 2:**
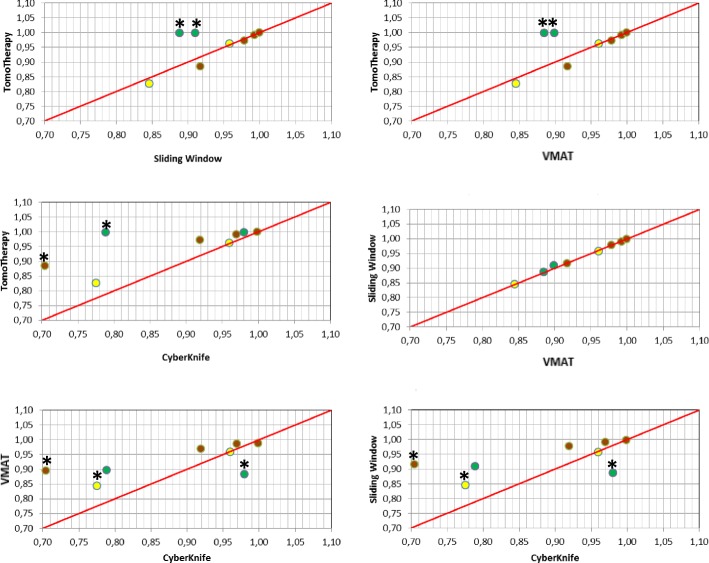
Pair-wise statistical dosimetric comparison between radiation techniques. The differences in sparing of OAR are shown at V18 Gy - V36.25 Gy for rectum and urinary bladder, and V14.5 Gy for femoral heads. Urinary bladder is indicated in yellow, rectum in brown, femoral heads in green. *- significant difference in dose distribution to OAR between radiation techniques

We assessed the dosimetric values for PTV and OAR from the patient-averaged DVH (these values are presented in further detail in Table 4). The mean prescription volumes did not show any relevant volumetric differences between the radiation techniques and varied between 90 cm^3^ and 160 cm^3^. The PTVV80 was found significantly lower for CK compared to other IMRT techniques (*p* = 0.04), whereas D2% and D98% values did not significantly vary between the techniques (Table 4). The patient-averaged DVH revealed statistically superior rectum sparing by IMRT techniques at the doses range of V18 Gy -– 32.6 Gy compared to CK technique (*p* < 0.05) (Figs. 1 and 2, Table 4). The highest rectum shielding was received by SW at medium-to-high dose range (Figs. 1 and 2, Table 4). The whole urinary bladder experienced the significant shielding by SW and VMAT at 18 Gy (V18 Gy, *p* < 0.05 versus CK) (Figs. 1 and 2; Table 4). No relevant dosimetric difference in the dose distribution to rectum and urinary bladder was found between techniques at V36.25 Gy. A significantly superior sparing of femoral heads was received by HT (V14.5 Gy). The CK affected mostly the left femoral head, for that spared highly the right femoral head (Figs. 1 and 2; Table 4).

**Table 4:** Averaged dosimetric values for PTV and organ at risk

	HT Mean ± SD	SW Mean ± SD	VMAT Mean ± SD	CK Mean ± SD
PTV				
V80 (%)	99.03 ± 0.63*	99.15 ± 0.59*	99.35 ± 0.44*	96.84 ± 2.48*
D2% (Gy)	44.10 ± 0.72	45.41 ± 0.69	45.17 ± 0.26	44.08 ± 0.39
D98% (Gy)	36.90 ± 0.41	36.86 ± 0.46	37.10 ± 0.27	35.82 ± 0.92
Urinary Bladder				
V18 Gy (%)	17.30 ± 12.88	15.46 ± 12.33*	15.52 ± 12.48*	22.51 ± 17.64*
V36.25 Gy (%)	3.70 ± 3.49	4.24 ± 4.11	3.98 ± 3.75	4.04 ± 3.82
Rectum				
V18 Gy (%)	11.51 ± 3.87*	8.34 ± 3.13*	10.40 ± 4.45*	29.62 ± 13.79*
V29 Gy (%)	2.72 ± 1.46*	2.18 ± 1.30*	2.99 ± 1.91*	8.11 ± 5.03*
V32.6 Gy (%)	0.72 ± 0.59	0.82 ± 0.69	1.29 ± 1.11	3.10 ± 2.24
V36.25 Gy (%)	0.01 ± 0.03	0.08 ± 0.11	1.22 ± 3.70	0.19 ± 0.25
Femoral head right				
V14.5 Gy (%)	0.05 ± 0.13*	11.21 ± 13.78	11.59 ± 18.60	2.04 ± 3.67
Femoral head left				
V14.5 Gy (%)	0.07 ± 0.18*	9.07 ± 11.04	10.16 ± 13.35	21.16 ± 13.75*

*Abbreviations*: *HT* Helical tomotherapy, *SW* Sliding Window, *VMAT* Volumetric Modulated Arc Therapy, *CK* Cyber Knife, *PTV* Planning target volume, *SD* standard deviation, *Dx (Gy)* Dose (Gy) absorbed by the certain percent (%) or absolute volume (cc) of the countered structure, *Vx* Percent of OAR volume exposed to certain radiation dose; *- Values with statistically significant difference (*p* < 0.05)

The calculated mean NTCP values and the model parameters for the rectum and urinary bladder are.

listed in Table 5. The HT revealed significantly superior mean NTCP values for rectum (NTCP = 2.3%, *p* < 0.05) than was demonstrated by VMAT (NTCP = 4.5%), SW (NTCP = 3.8%) and CK (NTCP = 5.8%). For urinary bladder the mean NTCP values were not significantly lower for SW (NTCP = 1.8%) compared to VMAT (NTCP = 2.5%) HT (NTCP = 2.8%) and CK (NTCP = 2.2%). Consequently, the HT demonstrated a lower NTCP outcome compared to CK or IMRT techniques for rectum and the NTCP for the urinary bladder showed no significant advantages for any technique.

**Table 5:** Calculated mean NTCP values (%) of all patients

Technique/Organ	Rectum	Urinary bladder
HT	2.3*	2.8
SW	3.8	1.8
VMAT	4.5	2.8
CK	5.8	2.2

*Abbreviations*: *HT* Helical tomotherapy, *SW* Sliding Window, *VMAT* Volumetric Modulated Arc Therapy, *CK* Cyber Knife; * - value with statistically significant difference (*p* < 0.05)

## Discussion

Our study evaluated a dosimetric comparison between IMRT-based techniques and robotic-assisted CK system by applying the SBRT for low risk prostate carcinoma. To our knowledge, this is the first time, when the NTCP parameters, which demonstrate probability of late rectal and urinary bladder radiation-related complications, were comparatively analyzed for SBRT techniques. Previous reports showed a high rate of biochemical, disease-free survival, along with an acceptable toxicity profile, with a larger fraction dose by applying SBRT techniques [9, 32–35]. Most single-center studies have used the CK technology demonstrating the feasibility of CK-based prostate SBRT [9–13, 33, 35–38]. McBride et al. demonstrated in their first multi-institutional Phase I study, an effective and safe use of hypofractionation with a CK System, by applying a 7.25–7.5 Gy fraction dose delivered in 5 fractions for the treatment of low-risk prostate adenocarcinoma [12]. The Prospective Randomized Phase III study, “PACE,” was developed to evaluate a clinical outcome following the SBRT monotherapy with CK, and further examined this therapy in comparison to surgery and conventionally fractionated IMRT in localized prostate carcinoma (http://www.clinicaltrials.gov/ct2/show/NCT01584258?term=PACE&rank=12). Unlike standard IMRT a technique, the CK technology performs an intrafractional matching of the beam targeting the prostate when motion is detected achieving the targeting errors of less than 1 mm [39, 40]. However, the longer treatment time with CK may result in intrafraction dose uncertainty because of bladder and bowel motion with anatomical deformation during the radiation treatment. Thus, Reggiori et al. showed that dose uncertainties for the targets and rectum amplified with the increase of time in patients treated with VMAT [41]. The mean treatment time that we observed for IMRT techniques, especially for VMAT and SW, was significantly less than for CK, (6 and 5 min compared to 42 min, respectively). The short treatment time helps to avoid dosimetric uncertainties in the target volume caused by bladder and bowel intrafraction form variation. Most treatment planning systems used for CK do not have advanced algorithms for the reducing of the planning time. Rossi et al. have proposed an automated treatment plan generation by using of “Erasmus-iCycle” optimizer for the creation of a beam angle class solution for noncoplanar prostate SBRT with CK to replace time-consuming beam angle optimization for each individual patient [16]. Using the in-house developed optimizer, the authors established 15-, 20-, and 25-beam class solutions without significant loss in plan quality compared to individualized beam angle selection, reducing the computation time for the plans generation by a factor of 14 to 25. Thus, using beam angle class solution instead of individualized beam angel selection, 25-beam plans could be generated in 31 min compared with 13 h.

We have demonstrated that both CK and IMRT-based techniques achieve similar dosimeteric outcomes, concerning PTV coverage, as well as providing highly conformal dose distribution. However, PTV homogeneity was significantly lowered in the CK treatment plans compared to rotational IMRT approaches. In addition, the IMRT techniques provided lower rectum and urinary bladder exposure at medium-to-high dose ranges than CK. Our findings are in agreement with the results obtained by MacDougall et al. [13]. Their results were provided from a dosimetric analysis gathered by comparing dose distribution between the CK and a VMAT with delivery of 35 Gy to the prostate in 5 fractions. The dose constraints for OAR were achieved by both techniques, however, PTV homogeneity as well as the mean planning and delivery time were in favor of VMAT. Furthermore, the use of VMAT was found to be superior when sparing OAR at lower radiation doses. Similarly, Lin et al. showed that applying 37.5 Gy in 5 fractions revealed superior PTV coverage and better rectum sparing at low doses with VMAT plans than with CK plans, although 6 MV photon beams were used for the VMAT treatment plans as opposed to 15 MV in our analysis [14]. Moreover, the VMAT plans demonstrated an excellent dose conformity resulting in faster dose falloff compared to CK plans. Finally, the author observed with VMAT plans fewer low-dose area, lower Monitor Units (MU), and faster delivery time than with CK plans. The authors speculated that the overall risk of secondary malignancy might be higher for CK through greater involvement of normal tissue receiving low RT dose, as well as higher MUs and treatment delivery time. Dong et al. comparatively analyzed the dose distribution for prostate SBRT (40 Gy in 5 fractions) by using of optimized robotic non-coplanar RT, termed 4π therapy, which is established on C-arm LINAC platform, and 2-arc VMAT [15]. Both planning methods demonstrated adequate PTV coverage. However, the 4π plans achieved significantly superior sparing of anterior rectum wall and penile bulb, reducing the maximum doses ad V50%, V80%, V90% and D1 cm^3^. The bladder dose was only slightly reduced by using of 4π therapy. Thus, by optimizing beam angles and fluences in the non-coplanar solution space, the authors have achieved superior quality for prostate SBRT compared to advanced VMAT plans. Rossi et al. developed systems for automatic generation of clinically deliver-able plans for robotic SBRT (autoROBOT). The quality of these plans was compared with VMAT plans that were also automatically generated, by applying of 9.5 Gy in 4 fractions [17]. Interestingly, in the autoROBOT and autoVMAT comparison with 3 mm PTV margins for all techniques, rectum doses (D1 cm^3^ and Dmean) was significantly lower in autoROBOT plans, with comparable PTV coverage and other OAR sparing. Compared to manual sparing, autoROBOT significantly improved rectum and urinary bladder sparing (D1 cm^3^ and Dmean), with equal PTV coverage. Thus, in contrast to results observed in our study by the comparison of manually generated VMAT and CK plans, authors demonstrated a superiority of non-coplanar robotic SBRT compared to coplanar VMAT when using the autoplanning for both techniques.

Assuming the same dose objectives for treatment planning, we can explain differences in dose distribution within PTV and OAR by the impact of radiation technique and by different dose calculation algorithms. The Multiplan planning system used for CK is less sensitive to dose constraints than the planning systems used for rotational approaches. Lowered sensitivity can result in the difference of PTV homogeneity in CK plans while using the same dose objectives in the planning system for IMRT techniques. However, a crucial factor that determinates the feasibility of radiation treatment plans is the optimization of dose constraints in each individual case.

Varying selection criteria, as well as differences in the target volume definition and dose constraints for OAR that do exist, actually describe the prostate SBRT. The difference in a cumulative radiation dose (between 33 Gy and 38 Gy), as well as in RT regimens (4 to 5 fractions) lead to substantial variations in an applied BED. Similarly, there is a wide spectrum of dose constraints for PTV and OAR in the available literature date [2, 7–9, 42–44]. For this reason, we used a combination of constraints from the PACE study and those recommended by Accuray and Varian Centers which deliver SBRT with CK/tomotherapy and RA/Sliding Window, consecutively. We assessed the BED by using the α/β value of 3 for rectum and 6 for urinary bladder to convert all constraint dose to 2 Gy per fraction. This allowed for an appreciation of the used dose objectives, according to criteria proposed by QUANTEC reports, which establishes the conventionally fractionated RT [19, 20]. Concerning the urethral sparing, is suggested that more heterogeneous dose distribution may provide a requisite prostatic urethra sparing within PTV. We restricted the maximum dose to 110% of the prescription dose in the treatment plans for all techniques to reduce the irradiation dose for the prostatic urethra. In the aspect of urethral toxicity, a multicentric Phase II study, which evaluates the SBRT in prostate cancer delivered by VMAT, including urethral sparing, is ongoing (http://www.clinicaltrials.gov/ct2/show/NCT01764646?term=NCT01764646&rank=1). Generally, the dose objectives for the prostate SBRT should be based on the datasets of the advanced radiotherapy technologies and large prospective randomized trials.

Another concern is the safety margins in the PTV delineation, which should be used to deliver SBRT for low-risk prostate carcinoma, to achieve a validated dosimetric comparison we used the same safety margins in both the CK and IMRT techniques, with a 3 mm margin in the dorsal direction and 5 mm margin in ventral and lateral directions. The use of image-guided RT with prostate verification immediately before treatment may not be enough for the precise delivery of radiation dose due to intrafraction prostate motion. According to established literature, the standard deviation of systematic and random errors due to intrafraction prostate motion varies from 0.2 to 1.7 mm and 0.4 to 1.3 mm, respectively [41, 45–49]. Considering that CK performs the real-time tracking of intrafractional prostate motion with a 1 mm precision in radiation dose delivery, MacDougall et al. have suggested the use of a safety margin of 3 mm for all directions in CK and 5 mm in VMAT [13]. Some authors observed a good clinical response as well as very low risk of intestinal grade 4 and 3 adverse effects applying the CK-based SBRT for low-risk prostate carcinoma by using of 2 or 2.5 mm safety margins into the rectal directions [50, 51]. Similarly, in the newly initiated prospective observational bi-center trial “HYPOSTAT”, the PTV was delineated with posterior margins of 2 mm for the CK-based SBRT [52]. On the other side, in the large series of reports describing the stereotactic RT with CK for localized prostate carcinoma, was observed a low rate of transient grade 3 and 2 urinary and rectal toxicities by using of 3 mm dorsal PTV margin and 5 mm safety margins in all other directions [41, 45–49]. Considering the highest exposure of rectum and urinary bladder for CK compared to IMRT that was revealed in this study, we would recommend reducing the PTV margins up to 2 mm in all directions for the CK-based SBRT, to minimize the risk of urinary and rectal toxicities as well as to provide a dosimetric advantage compared to advanced IMRT techniques.

One lingering question in regards to SBRT for prostate cancer is the RT regimen. King et al. reported on the fourfold reduction in Grade 1 urinary toxicity and a sevenfold reduction in Grade 1 rectal toxicity in favor of the every-other-day SBRT compared to daily SBRT consisted of 36.25 Gy in 5 fractions [9]. The authors, ultimately, recommended treating the prostate with an every-other-day dose schedule in order to allow the SBRT to minimize late effects in normal tissue. However, only a randomized trial would be able to properly study differences between different RT regimens.

We focused on analyzing the probability of late rectal and urinary toxicities by using SBRT on low-risk prostate carcinoma. Despite the significant dosimetric advantage in rectum protection for SW (Table 4), the NTCP values of late rectal toxicities reveal that HT is superior in this regard (Table 5). Use of fixed-fields IMRT, but not rotational techniques, was shown to improve the NTCP parameters for urinary bladder. The SW or HT, but not VMAT, generally are preferred in order to reduce the probability of late rectal in the treatment of low-risk prostate carcinoma. The NTCP for the urinary bladder showed no significant advantages for any technique.

This study is based on the treatment planning systems Eclipse™10, Tomo planning system version 5 and the Multiplan® planning system version 5.2. In the development process of this work the treatment planning systems were routinely used in our institute. The newer algorithms for the optimization lead into a different way of planning. For example the new Photon Optimizer in the Eclipse™15 has an improved OAR and target overlap modeling. With similar dose objectives in the planning process the resulting dose distribution and DVH differs slightly between Eclipse™10 and Eclipse™15. Therefore, the individual optimization of dose objectives may improve the target coverage and OAR sparing by using of Eclipse™10 treatment planning system. The NTCP values are based on DVH results so a newer algorithm could show a different result. There are some studies about the influence or impact of dose calculation algorithms on NTCP values, especially for lung cancer [53–55]. Because of a steady progress of algorithms to increase the accuracy of dose distribution and to minimize uncertainties, an additional work could investigate the impact of an update of the Eclipse™10 to Eclipse™15 regarding the NTCP of prostate SBRT.

Our study is limited by its retrospective nature and small number of study population which precludes big conclusions and planning’s parameters used should not be extrapolated for all cases. For example, the PTV margins for prostate SBRT should be defined based on radiation technique used, carcinoma stage and prostate volume. Another possible limitation is selection bias due to large difference in the prostate and rectal volume in analyzed patients (Additional file 1: Table S1. For this reason, the estimated treatment plans demonstrated large variations in the values of PTV coverage and OAR sparring between patients. In addition, the dose constraints for rectum and urinary bladder recommended by QUANTEC are based on 3-D conventional RT datasets. The advanced IMRT and CK techniques provide highly conformal dose distribution, performing superior OAR sparing compared to 3-D CRT, thus the dose constraints for OAR must be adopted for advanced IMRT and CK techniques used for prostate SBRT. Regarding NTCP analysis, we used the Lyman’s model of rectal and bladder toxicities for estimating of NTCP values. However, Viswanathan et al. aver that no convenient quantitative model exists, which can satisfactorily analyze late bladder toxicity after external beam radiotherapy [20]. This is due to lack of a clear dose response and functional variability of the bladder. Finally, the radiation plans can be optimized by individual modification of dose objectives for each treatment case. This argument can diminish the relevance of the obtained results, despite the use of similar dose objectives in the estimation of the radiation plans. Thus, selection criteria for dosimetric comparison between different radiation approaches should be further optimized.

## Conclusion

This analysis is focused on the evaluation of the dosimetric feasibility of different SBRT techniques in the therapy of low-risk prostate cancer. For all techniques we applied the same safety margins for the delineation of the target volume as well as the same dose objectives were used for the plan optimizations. Major findings to emerge from this study are as follows: (i) All techniques showed a high conformal dose distribution in achieving OAR constraints; (ii) The CK revealed lower homogeneity within the target volume; (iii) The CK revealed the highest exposure of rectum and urinary bladder compared to IMRT, especially at medium-to-high dose ranges; (iv) comparing IMRT techniques, the SW displayed superior rectum sparing at medium-to-high dose range, whereas both SW and VMAT revealed superior bladder sparing; (v) techniques such as SW or HT — but not VMAT — demonstrate a reduced probability of late rectal complications; (vi) The mean treatment delivery time was significantly less for IMRT techniques than for CK, with shorter mean values for SW (6 min) and VMAT (5 min) compared to 42 min for CK. Generally, this dosimetric analysis revealed a higher protection of the rectum and bladder by using IMRT techniques compared to CK for the SBRT of prostate cancer. However, considering the possible optimization of radiation plans by the individual modification of dose objectives for each case, the radiation technique for prostate SBRT should be selected individually dependent on the treatment strategy.

## Additional file

Additional file 1: **Table S1.** Variables for patients’ population. (DOCX 12 kb)

## Data Availability

The datasets supporting the conclusions of this article are available in the repository of the Department of Radiotherapy and Radiooncology, University Hospital of Muenster.

## Abbreviations

BED: Biological equivalent dose
CBCT: Cone beam computed tomography
CK: Cyber Knife
CTV: Clinical target volume
DVH: Dose-volume histograms
HT: Helical tomotherapy
IMRT: Intensity-modulated radiotherapy
LINAC: linear-accelerator
LKB: Lyman-Kutcher-Burman model
MRI: Magnetic resonance imaging
NCCN: National Comprehensive Cancer Network
NTCP: Normal tissue complication probability
OAR: Organ at risk
PFS: Progression free survival
PTV: Planning target volume
QUANTEC: Quantitative Analyses of Normal Tissue Effects in the Clinic
SBRT: Stereotactic body radiotherapy
SW: Sliding Window
TCP: Tumor control probability
VMAT: Volumetric Modulated Arc Therapy
