# Supplementary material for: A treatment planning study comparing IMRT techniques and cyber knife for stereotactic body radiotherapy of low-risk prostate carcinoma
Source: Radiat Oncol. 2019 Aug 9;14:143. doi: 10.1186/s13014-019-1353-6 (PMC6689170; doi:10.1186/s13014-019-1353-6)
Supplement: Supplementary file 1 — Table S1. Variables for patients’ population. (DOCX 12 kb) [file 13014_2019_1353_MOESM1_ESM.docx]

| Characteristics | n (%) |
| --- | --- |
| No. of patients  Median age/range | 27 (100%)  68y /58y - 77y |
| ECOG 0/1 | 16 (60%) / 11 (40%) |
| Median pre-Tx PSA/range, ng/mL | 6.05 / 2.7-9.4 |
| T stage |  |
| T1c | 20 (75) |
| T2a | 7 (25) |
| Gleason Score |  |
| 3+3=6 | 22 (80) |
| 3+4=7a | 5 (20) |
| Median Prostate volume/range, cc | 31 / 27-58 |
| Median rectal diameter/range, cm | 5 / 3.4-7.4 |
| Median bladder volume/range, mL | 300 / 220-410 |
| IPSS Score |  |
| 0-7 | 17 (63) |
| 8-19 | 7 (26) |
| 20-35 | 3 (11) |
| Hormon treatment +/- | 2 (7) / 25 (93) |
| Nicotine abuse +/- | 5 (25%) / 15 (75%) |
| Alcohol abuse +/- | 2 (10%) / 18 (90%) |

**Table 1. Variables for patients` population**
